# Sample Size Estimation for Detection of Splicing Events in Transcriptome Sequencing Data

**DOI:** 10.3390/ijms18091900

**Published:** 2017-09-05

**Authors:** Wolfgang Kaisers, Holger Schwender, Heiner Schaal

**Affiliations:** 1 Department for Anaesthesiology, Heinrich Heine University, 40225 Düsseldorf, Germany; 2 BMFZ, Heinrich Heine University, 40225 Düsseldorf, Germany; schwender@math.uni-duesseldorf.de (H.S.); schaal@uni-duesseldorf.de (H.S.); 3 Mathematical Institute, Heinrich Heine University, 40225 Düsseldorf, Germany; 4 Institute for Virology, Heinrich Heine University, 40225 Düsseldorf, Germany

**Keywords:** splicing, RNA-seq, transcriptome sequencing, alternative splicing, *wgis*

## Abstract

Merging data from multiple samples is required to detect low expressed transcripts or splicing events that might be present only in a subset of samples. However, the exact number of required replicates enabling the detection of such rare events often remains a mystery but can be approached through probability theory. Here, we describe a probabilistic model, relating the number of observed events in a batch of samples with observation probabilities. Therein, samples appear as a heterogeneous collection of events, which are observed with some probability. The model is evaluated in a batch of 54 transcriptomes of human dermal fibroblast samples. The majority of putative splice-sites (alignment gap-sites) are detected in (almost) all samples or only sporadically, resulting in an U-shaped pattern for observation probabilities. The probabilistic model systematically underestimates event numbers due to a bias resulting from finite sampling. However, using an additional assumption, the probabilistic model can predict observed event numbers within a <10% deviation from the median. Single samples contain a considerable amount of uniquely observed putative splicing events (mean 7122 in alignments from TopHat alignments and 86,215 in alignments from STAR). We conclude that the probabilistic model provides an adequate description for observation of gap-sites in transcriptome data. Thus, the calculation of required sample sizes can be done by application of a simple binomial model to sporadically observed random events. Due to the large number of uniquely observed putative splice-sites and the known stochastic noise in the splicing machinery, it appears advisable to include observation of rare splicing events into analysis objectives. Therefore, it is beneficial to take scores for the validation of gap-sites into account.

## 1. Introduction

Current analysis strategies for detection of splicing events mostly consider isoform expression [1,2,3]. As isoform identification in complex genomes currently suffers from insufficiencies [4]—the detection of alternative splicing is associated with low sensitivity—especially when transcript abundance or read coverage is low [5,6]. Therefore, a reasonable alternative strategy is to focus on gapped alignments, an approach we elaborated recently [7].

Genomic alignments of reads obtained from whole transcriptome sequencing contain gapped alignments due to the removal of introns during pre-mRNA splicing. To increase the detection sensitivity of gapped reads resulting from low abundant transcripts, alternative splicing, or sporadically used splice-sites, data from multiple samples needs to be merged. Gap-sites are alignment gap locations possibly shared by multiple alignments. They represent putative splice-sites and need to be validated because they are reported by aligners with a high false discovery rate (FDR) [7].

### Observation of Splicing Events

For the detection of splicing events, we recently developed three R packages allowing accumulative extraction of gap-site information from transcriptome sequencing data [8], calculation of two scores for gap-site validation (Gap Quality Score, *gqs* and Weighted Gap Information Score, *wgis*), and annotation of gap-sites [7]. For each gap-site, detected in a batch of samples, the total number of covering alignments in all samples (nAligns, alignment depth) and the number of samples in which a gap-site was identified (nProbes, multiplicity) are reported.

The distribution of *wgis* values implies a division of gap-sites into four gap-quality levels (*gql*0 = not validated to *gql*3 = high confidence level). Thus, gap-sites are a heterogeneous population with varying alignment coverage and multiplicity as well as varying resemblance to confirmed splicing events.

Gap quality level 0 is assigned to a gap-site when the value of *wgis* is 0. The value wgis = 0 indicates that either the number of matching nucleotides in the merged samples (qsm< 16) or one of the MaxEnt scores (score5 or score3) are below threshold. Thus, evidence from alignments is sparse or splice-site strength is too low for validation. A detailed description can be found in our recently published manuscript [7].

## 2. Results

### 2.1. Sample Size Estimation

The starting point of considerations on sample size estimation is the expectation that the number of observed gap-sites should increase with the number of samples in a batch. The number of observed gap-sites thus was examined by repeatedly drawing sample batches of varying size from the fibroblast transcriptomes.

#### Number of Gap-Sites Observed in Small Samples

**Figure 1:**
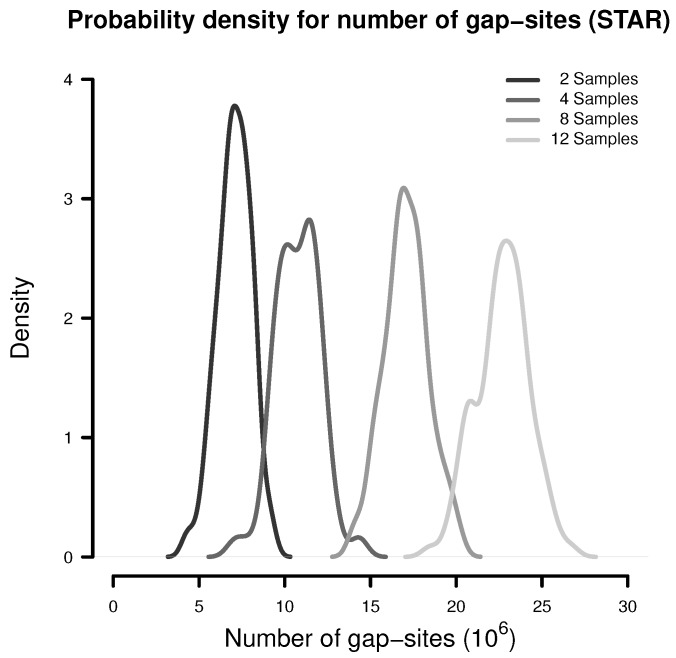
Number of gap-sites observed in samples of different sizes (STAR).

In a simulation experiment, 100 random batches consisting of 2, 4, 8 and 12 samples were extracted (from the 54 fibroblast samples) and analysed for the presence of gap-sites as expected from alignments by STAR. The probability density estimates shown in Figure 1 indicate, that the number of identified gap-sites increase with sample size.

A more detailed analysis, however, shows that the increase in observation numbers is not equally distributed between gap-sites of different gap-quality levels. The number of not validated (*gql*0) gap-sites increases nearly linearly with a rate of 128,000 new gap-sites per sample, while the number of *gql*3 sites increases only at a much lower rate (Table 1).

Thus, gap-sites are a heterogeneous population with varying contributions to growing total number of observations in larger samples. An alternative interpretation of these varying contributions as consequence of varying observation probabilities leads to the hypothesis that a calculation of expected values from observation probabilities provides a prediction of total event numbers. In the following, we elaborate and evaluate this model.

### 2.2. The Probabilistic Model for Prediction of Event Numbers

The probabilistic model for observation of events (identification of gap-sites in a sample or in a batch of samples) is based on two assumptions: The observation of gap-sites in single samples are

- random events
- independent from each other

Independence means, that the observation probabilities for the observation of gap-sites are not influenced by observations in other samples or by observations of other gap-sites.

Using these assumptions, a relation between observation probability in single samples and in a batch of 54 samples can be related using basic probabilistic considerations. In essence, the considerations are based on the fact that not observing a gap-site in a batch of samples is equivalent to not observing a gap-site in any sample in the batch.

#### 2.2.1. Definition of Two Probabilities

In the probabilistic model, two types of probabilities are considered: the observation probabilities ($p_{j}$) and the observation prior (Π).

The observation probabilities represent the gap-site multiplicity (the number of samples in which a gap-site is identified) in the model.

As a real batch consists of a finite number of samples (*n*), the observation probabilities are numbers in $\left\{\frac{1}{n},\frac{2}{n},\ldots ,1\right\}$.

**Figure 2:**
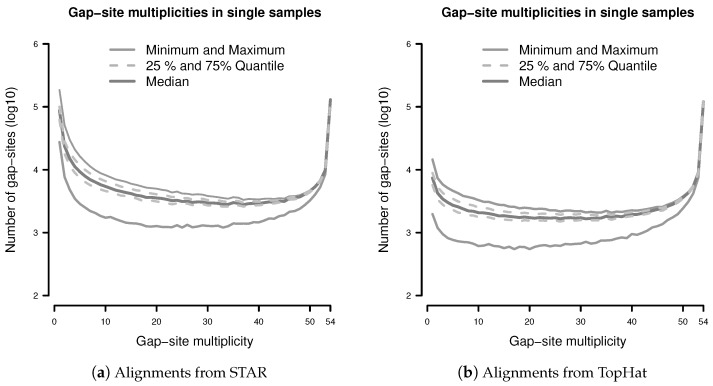
Distribution of gap-site multiplicities in single samples: (**a**) Alignments from STAR; (**b**) Alignments from TopHat. For each gap-site, the multiplicity in the whole batch of 54 samples was determined. Then, for each of the 54 samples, the absolute number of multiplicities contained therein was tabled. Median gap-site numbers (dark gray) together with 25% and 75% quantiles (dashed lines) in 54 samples are shown. The light gray lines indicate minimal and maximal number of gap-sites.

The observation prior represents the relative abundance of gap-site multiplicities in real samples. The distribution of absolute numbers of gap-site multiplicities is shown in Figure 2. The U-shaped distribution indicates the fact that 73.7% (in TopHat) and 90.3% (in STAR) of gap-site multiplicities are <5 or >50 and thus are located at the extremes. Also, there is considerable variation between different samples (SD/mean > 34% for nProbes < 10), which is also demonstrated in Appendix C.

Normalising both axes in Figure 2 creates the observation prior (Π).

#### 2.2.2. Calculation of Expected Values

The two probabilities are connected to each other in a two step model, in order to model the observation of a single gap-site in a single sample: when a gap-site is to be observed, first an observation probability (*p*) is drawn from the observation prior Π. Then, the observation is drawn from a binomial distribution with probability *p*.

The expected number of gap-site observations in a sample of size ν then can be calculated by an integration. As real sample batches consist of a finite number of samples, both probabilities are discrete and thus expectations are calculated using sums. In a batch of size ν, the expected number of observed gap-sites ($\hat{|S_{\nu }|}$) is calculated from the probabilistic model as

(1) $$\begin{array}{c} \hat{|S_{\nu }|}=\left|S_{n}\right|-\sum_{j=1}^{n-1}{\left(1-\frac{j}{n}\right)}^{\nu }z_{j}, \end{array}$$

where $|S_{n}|$ is the number of gap-sites in the full batch (of size *n*) and $z_{j}$ is the total number of gap-sites with multiplicity *j* therein. The detailed definitions and calculations are shown in Appendix B.

### 2.3. Evaluation of the Probabilistic Model

**Figure 3:**
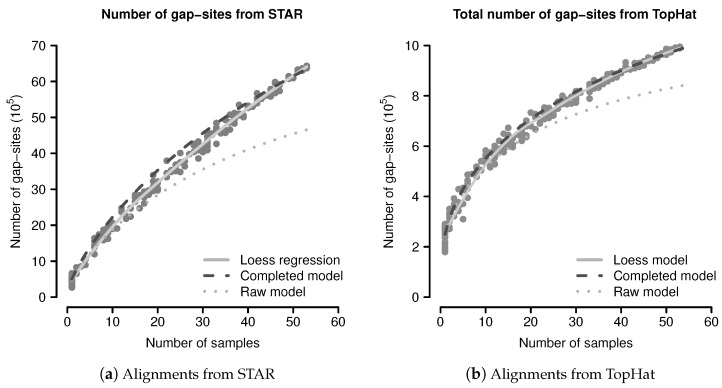
Observed and predicted numbers of gap-sites: (**a**) Alignments from STAR; (**b**) Alignments from TopHat. Observed number of gap-sites (y-axis) from 200 randomly drawn sub-batches with varying numbers of samples (x-axis) are shown as solid circles (dark gray). Results from a Loess regression are shown as solid line (light gray). The predictions from the (uncorrected) raw model indicate a too small terminal slope (dotted line). The predictions from the completed model indicate improved consistency with the observed numbers.

The predicted and observed numbers of gap-sites were evaluated using a simulation study. From 54 sequenced fibroblast transcriptomes, 200 random sub-batches of random size (drawn from a uniform distribution on {2,…,53}) were extracted and completely analysed for number of gap-sites. The data from all single samples were added to the simulated data. The number of observed events are shown in Figure 3. The mean number of gap-sites is modelled by a Loess regression (solid line).

#### Limitations Arising from Finite Samples

The predicted number of observed events using Equation (1) are considerably lower than the observed numbers, mainly due to a too low terminal slope that can be seen in the predictions from the raw model (dotted line) in Figure 3. Considerations shown in the model evaluation (Appendix B.2.3) clarify that a too small terminal slope is caused by an underestimated number of rare events (for example unique gap-sites). The following, this effect is related to estimation from finite samples.

The observation probabilities for gap-sites, displayed in Figure 2, show sharp maxima at both ends of the *x*-axis (observation probabilities near 0 and 1). Due to steep ascents, estimation accuracy relies on data from close proximity to the extremes. However, due to finite sample sizes, data on the proximity of the extremes is limited.

The resulting impact is quantified by examination of events (gap-sites) with multiplicity 1 (*unique* events). When a batch of finite size *n* is analysed, the lowest observable multiplicity is 1, assigned with an observation probability of $\frac{1}{n}$. The observation probability of unique events approaches 0 with increasing *n* and thus the probability of being unobserved should be near 1. However, according to Equation (1), the probability of being unobserved is ${\left(1-\frac{1}{n}\right)}^{n}$ for unique events and thus the theoretical limit (approached with <1% error for n=54) is

(2) $$\begin{array}{c} \lim_{n\to \infty }{\left(1-\frac{1}{n}\right)}^{n}=e^{-1}\approx 0.368. \end{array}$$

Consider the number unique events ($m_{u}$) in batch of *n* samples. As a consequence, the predicted number of unique events from Equation (1) is

$$\begin{array}{cc} m_{u}\;\left(1-\frac{1}{e}\right) & \approx \;m_{u}\times 63.2\% \end{array}$$

in a batch of size *n* (instead of $m_{u}$). Thus, the number of unique events in the full sized batch is 36.8% underestimated—an inaccuracy that cannot be avoided by increasing sample size.

### 2.4. Correction for Estimation Inaccuracies

As the lack of information at the extremes does not inevitably distort available data, we explored whether the model predictions recover when the informational gap is closed by adding artificial estimations.

Thus, virtual events with multiplicity <1 are added to the data, which does not change the relations between observed gap-site multiplicities, and is outside a range accessible by real samples. The observation probabilities are recalculated after adding $7.75\times 10^{6}$ gap-sites with multiplicity 0.24 to alignments from STAR and adding $4.5\times 10^{5}$ gap-sites with multiplicity 0.4 to alignments from TopHat. These numbers were determined by manually optimising the total number of predicted events in the full sized batch (*n* = 54).

#### Predictions by the Completed Probabilistic Model

The event numbers predicted by the completed model and the observed event numbers are shown in Figure 3, where the predictions of the completed model are shown as dashed dark line. The median difference between the corrected model and the mean values calculated by Loess regression is 8.16% in alignments from STAR and 1.93% in alignments from TopHat. The probabilistic model thus provides a sufficient explanation for the observed gap-sites numbers.

### 2.5. Basal Rate for Observation of Gap-Sites

The number of gap-sites modelled by the Loess regression in Figure 3 show a nearly linear increase in batches of large size (>40 samples). This constant terminal slope defines a basal rate for the observation of gap-sites (*gbr*), meaning that with every added sample, the total number of gap-sites increases by a constant value.

As, in an empirical sample, all unique events are part of the *gbr*, the *gbr* must be greater than the mean content of unique events in each sample. The *gbr* is 8056 gap-sites per sample in alignments from TopHat and 92,764 gap-sites per sample in alignments from STAR.

In alignments from TopHat, in total 384,576 unique gap-sites are identified with mean 7122 (SD 2905) per sample. In alignments from STAR, in total 4,655,597 unique gap-sites are identified with mean 86,215 (SD 34,464) per sample (Details are shown in Appendix C).

Thus, in the analysed samples, approximately 90% (88.4% in TopHat alignments and 92.9% in STAR alignments) of the *gbr* is represented by unique gap-sites.

### 2.6. Sample Size Estimation

The application of independency (presupposed in the probabilistic model) to calculation of sample sizes, required for experimental observation of splicing events (for example non-canonical splicing), allows utilisation of a simple binomial model. First, the lowest observation probability ($p_{o}$) for splice-sites of interest must be estimated. Together with the required power ($p_{w}$), the number of required samples can be calculated from the binomial model using the formula

(3) $$\begin{array}{c} n=\frac{ln(1-p_{w})}{ln(1-p_{o})} \end{array}$$

(details of derivation are shown in Appendix D). The required sample sizes for a selection of observation probabilities are shown in Table 2.

In order to provide a rule of thumb, recommended sample sizes for detection power of >80% are >10 for rare gap-sites ($p_{o}<0.15$), 3–10 for occasionally observed gap-sites ($0.15<p_{o}<0.5$), and 1–3 for regularly observed gap-sites ($p_{o}>0.5$).

For detection of non canonical splicing or alternative splicing, required sample sizes have been proposed in the range of ≈1–$4\times 10^{8}$ reads (per sample or condition) [6]. According to the described model (and assuming a power of 80% and $180\times 10^{6}$ reads per sample), this would suffice for detection of gap-sites with observation probabilities down to $\frac{1}{3}$.

As observation probabilities in single samples depend on alignment depth, calculated sample sizes need to be adjusted to alignment numbers (see Appendix A).

## 3. Discussion

The goal for the current investigation was to answer the question how many gap-sites are observed in batches of different sizes and to solve the problem of sample size estimation. The observation that gap-sites are a heterogeneous population differing by observation multiplicity and by validation status (*gql*) led to the construction of a (simple) probabilistic model. Using predictions from the model and a simulation study on observed data, the accuracy of the model is further explored.

Besides inaccuracies resulting from finite sampling (corrected by adding artificial estimates), the predicted and observed number of gap-sites are in good accordance (median deviation <2%) with TopHat alignments and in acceptable accordance (median deviation <10%) with STAR alignments.

Although the results do not provide a direct proof of the probabilistic model, we discuss the consequences arising from the model assumptions, namely (i) that gap-sites are (in range of detectable variation) observed independently from each other and (ii) the observation of gap-sites is a random event.

### 3.1. Independency of Gap-Site Observations

The independency implies that the likelihood of observation of single gap-sites is not influenced by previous observations or by the presence or absence of other gap-sites. As a regulated co-occurrence of gap-sites in a subset of samples would increase the variance of observed numbers, independency can only be deduced down to co-regulated gap-site clusters of size $10^{4}$ or more, which provides only a weak upper boundary. The situation for gap-sites is thus analogous to the practice in gene expression data, where the assumption of independent regulation also has been applied [9].

### 3.2. Observations of Gap-Sites Are Random Events

The view that gap-site observation is affected by random effects is consistent with the process of mRNA sequencing and with procedures in the alignment algorithms. We additionally propose, that random effects also are inherent in the splicing machinery.

#### Stochastic Noise in the Splicing Machinery

The high degree of evolutionary conservation [10] and the ubiquity of splicing [11,12] underline the functional relevance of the splicing process. Alternative splicing facilitates the generation of multiple transcript (mRNA) isoforms from single genes and thereby the production of ≈100,000 transcripts from ≈20,000 genes [13,14] in humans. The diversified transcript pool potentially expands protein functionalities, which may be advantageous for individuals (documented for a large variety of genes [15,16]) as well as for the species (by increasing the rate of evolutionary change [11,17,18,19]). The fact, that for almost all genes only a single translated (protein) isoform could be detected [20,21,22], and the existence of a subsequent (quality based) filter (for example NMD) [23,24,25] emphasise a functional role of the diversified transcript pool as a driver of evolutionary change. Evolutionary demands for variation may imply that splicing noise rather than splicing accuracy is under selection pressure. This could in turn explain why the splicing code is degenerated [26,27] and why complex splice regulatory mechanisms are necessary. Thus, the described stochastic noise in the splicing machinery [28,29,30] may not be accidental. This randomness would be in accordance with the probabilistic model described here. Analysis thus needs to separate three sources of stochastic variation: mRNA sequencing, alignment of sequencing data to the genome, and the splicing process itself. For this differentiation, accounting for splice-site strength will be helpful, which is included in our recently described *wgis* score [7].

### 3.3. Consequences of Basal Rates for Observation of Gap-Sites

The simulation data (Figure 3) indicates that for new gap-site observations, a constant basal rate exists even for larger batch sizes (n>40). This basal rate largely consists of unique gap-sites.

The alignments from STAR contain a very high number (4,655,597) of unique gap-sites. Additionally, the corrections introduced into the probabilistic model (although artificial) may be indicative of a much higher number of potential gap-sites required for the explanation of this data. Numbers of splicing events in the range of 5–$10\times 10^{6}$ are very high and potentially cannot be explained by noise in the splicing machinery alone. Also, 92.1% of unique gap-sites in STAR alignments are *gql*0-sites, meaning they are not validated by *wgis* and thus either there is only weak support from alignments or they are weak splice-sites. Therefore, the contribution of artificial sources to observation of *gql*0-sites in unique gap-sites reported by STAR may be not be negligible.

### 3.4. Observation of Gap-Sites under Different Experimental Conditions

#### 3.4.1. Influence of Read-Length and Alignment Depth

We assume, that aligned reads are randomly distributed on the transcriptome. As direct consequence, observation probabilities for splice-junctions are influenced only by alignment depth and not by read length.

Thus, variations in alignment depth primarily influence measures correlating with number of matching nucleotides. Also, likelihood of observation in a sample as well as likelihood of validation (by *gqs* or *wgis*) will perceivably change only when alignment depth crosses thresholds.

We consider, for instance, a reduction of alignment depth by 50% in alignments from STAR. In order to reach a 50% validation rate for gap-sites, 240 alignments are required when *gqs* is used and 19 alignments when *wgis* is used [7]. Thus, the validation status essentially will remain unchanged for gap-sites supported by 1000 alignments. Gap-sites supported by 300 alignments will presumably no more be *gqs*-validated (but still *wgis*-validated) and gap-sites supported by one alignment are likely to become unobserved. The example shows that heterogeneous effects of experimental conditions on observation probabilities are provoked.

These considerations show that spreading of sequencing power on more samples may be a sensible approach as long as observation probabilities in single samples is not impaired. Thereby, a more complete picture of the stochastic dispersion may be generated.

#### 3.4.2. Influence of Sequencing Technology

Meanwhile, significant advances have been made since the invention of second generation sequencing (SGS) [31,32]. Third generation sequencing (TGS) platforms, for example single molecule real-time sequencing technology (SMRT) [33] and nanopore sequencing [34], offer read lengths up to 20,000 base pairs but currently is associated with 60–100 higher costs per (Giga-) base than SGS on the Illumina platform [32]. As splice-site observation probability is not improved by longer reads and considerable amounts of samples are required for the detection of occasionally observed splice-sites, TGS unlikely will replace SGS here. Also, the splice-site detection mechanism in rbamtools meanwhile does not consider whether different gap-sites belong to the same transcript or arise from the same read. Though, observation probability may not be altered by short reads (for example read length = 20), the likelihood of gap-site validation is severely impaired as the minimal number of matching nucleotides (on both sides of the alignment gap) is limited (to 10 in the example).

#### 3.4.3. Influence of Different Species and Tissues

We suppose that the U-shaped pattern of gap-site multiplicities present in our fibroblast sample can be found in most tissues and species. Thus, the considerations of the shown probabilistic model should apply.

Gap-sites with high observation probability (the right hand side of the U-shape) are caused by genes ubiquitously expressed in a tissue. Gap-sites with low observation probability (the left hand side of the U-shape) are caused by splicing events that are only occasionally present in a tissue and also by errors in sequencing and alignment. A shift in the relation between gap-sites with high and low observation probability might be introduced by different tissues (for example via differing numbers of constitutively expressed genes). In order to obtain the exact distribution of observation probabilities, gap-site multiplicities on a batch of sufficient size will have to be analysed.

### 3.5. Comparison with Other Analysis Strategies

In a recent report, re-extraction of reads from two human samples that were sequenced with ultra-high coverage (≈$10^{9}$ reads), had been described [35]. In order to recover 80% of alternative splice events from the main sample, 100–$150\times 10^{6}$ were required and more than $400\times 10^{6}$ reads were required in order to recover 80% of differential alternative splicing events. According to the estimations presented here, these read numbers would suffice only for detection of regularly observed gap-sites ($p_{0}>0.5$).

Also, as transcriptome sequencing is commonly performed using 50–$100\times 10^{6}$ reads [6], the read numbers resulting from the sample size estimation presented here are much higher.

## 4. Materials and Methods

### Transcriptome Sequencing Data

All transcriptome data shown in this study originates from an investigation where the effects of age, gender, and UV exposition were studied in 54 samples of dermal fibroblasts obtained from healthy human donors. A main result of the study is that no consistent differential expression of genes is observed [36]. The gene expression in the 54 samples is thus deemed to be homogeneous. Collection and processing of dermal samples from donors was approved by the Ethical Committee of the Medical Faculty of the University of Düsseldorf (# 3361) (11 April 2011). Raw Fastq files are available under ArrayExpress accession E-MTAB-4652 (ENA study ERP015294). This batch of samples is used as training batch where statistical distributions are derived from.

The transcriptomes were sequenced on an Illumina HiSeq 2000 sequencer. Sequences were aligned against the Human genomic sequence (GRCh38) using STAR (version 2.4.1d modified) [37] and TopHat (version 2.0.14) [38,39] aligners. For alignment, the soft-masked version of the toplevel sequence regions were downloaded from ENSEMBL (version 76). As both aligners neglect effects of soft-masking, alignments on soft-masked and unmasked sequences yield equal results. The transcriptome sequencing data of the 54 fibroblast samples contain in mean $179.0\times 10^{6}$ alignments from STAR and $162.5\times 10^{6}$ alignments from TopHat (for more details see Appendix A).

The number of gap-sites present in single samples as well as in merged samples has been calculated using the framework provided by R packages rbamtools (version 2.16.0, available on CRAN), refGenome (version 1.7.3, available on CRAN), and spliceSites (version 1.23.3, available on Bioconductor).

## 5. Conclusions

The observation of sporadic splicing events, which, for example, may be due to splicing inaccuracies, can be a worthwhile analysis objective. Their observation can be described using a simple binomial model. High read numbers are required for their detection.

## Figures and Tables

**Table 1:** Number of gap-sites from different sample sizes (STAR).

nFiles	Total	*gql* = 0	*gql* = 3
2	706	378	92
4	1076	666	105
8	1708	1179	124
12	2270	1659	137

Absolute number of gap-sites (in 1000).

**Table 2:** Sample size calculation.

$p_{o}$	Sample Size
0.1	16
0.15	10
0.2	8
0.5	3
0.8	1

Required sample size for detection power of >80%.

**Figure A1:**
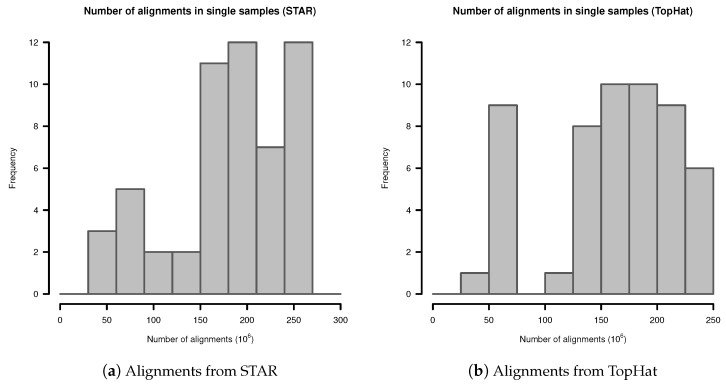
Number of alignments in transcriptome sequencing data of single samples: (**a**) Alignments from STAR; (**b**) Alignments from TopHat.

**Figure A2:**
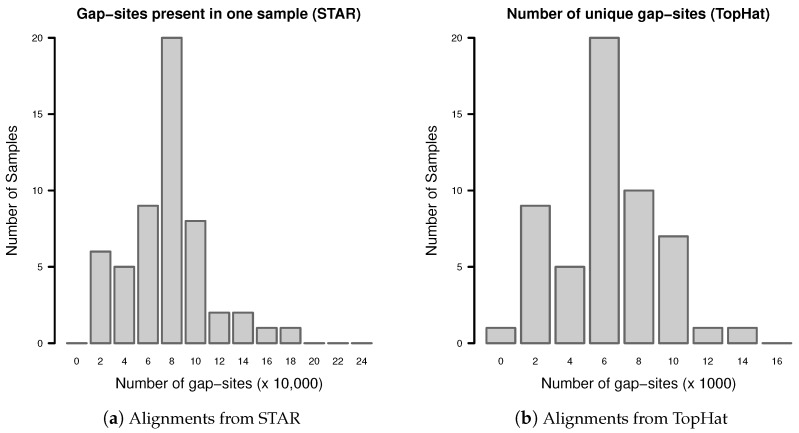
Number of unique gap-sites (gap-sites present in only one sample).

## Abbreviations

FDR	False Discovery Rate
nAligns	Number of supporting alignments (for a gap-site)
nProbes	Number of samples in which a gap-site is identified
*gqs*	Gap quality score
*wgis*	Weighted Gap Information Score
*mcl*	Minimum CIGAR length
*qsm*	Quartet sum of MCL
*nlstart*	Number of left start (positions)
SD	Standard deviation

## Appendix A. Number of Alignments in Fibroblast Transcriptome Samples

In single samples, alignments from STAR aligner contained in mean $179.0\times 10^{6}$ alignments (SD $62.1\times 10^{6}$). Alignments from TopHat in single samples contained in mean $162.5\times 10^{6}$ alignments (SD $58.0\times 10^{6}$). The detailed distribution is shown in Figure A1.

## Appendix B. Probabilistic Model for Observation of Events

### Appendix B.1. Definitions

#### Appendix B.1.1. Observation Probabilities

Let *G* be the set of events (observed gap-sites) $G=\{g_{j}|j\in 1,\ldots ,m\}$ (indexed by j) and |G|=m the total number of observable events. The value of *m* is not known exactly, because events might not be actually observed in any sample.

Consider a batch of samples, $B_{n}=\left\{s_{i}|\;i\in 1,\ldots ,s_{n}\right\}$ of size *n* ($|B_{n}|=n$), indexed by *i*. Define a sample $s_{i}$ as a set of observed events $s_{i}:=\left\{g_{j}:g_{j}\in s_{i}\right\}\subset G$, where $g_{j}\in s_{i}$ reflects the fact that event *j* has been observed in sample *i*. The indicator function reflects the observation of an event in a sample:

$$1_{i}\left(g_{j}\right):=\left\{\begin{array}{cc} 1 & \mathrm{when}\;g_{j}\;\in \;s_{i}\\ 0 & \mathrm{when}\;g_{j}\;\notin \;s_{i}. \end{array}\right.$$

Define, that an event $g_{j}$ is observed in a batch $B_{n}$, exactly when $g_{j}$ is observed in at least one sample $s_{i}$. By designation

(A1) $$\begin{array}{c} S_{n}:=\overset{n}{\underset{i=1}{⋃}}s_{i}\subset G, \end{array}$$

the observation of $g_{j}$ in $B_{n}$ is equivalent to $g_{j}\in S_{n}$. The cardinality of $S_{n}$ ($|S_{n}|$) is the number of observed events in $B_{n}$.

The expected number of observed events will be calculated using the opposite formulation of Equation (A1), obtained using De Morgan’s law

(A2) $$\begin{array}{c} S_{n}^{c}=\overset{n}{\underset{i=1}{⋂}}s_{i}^{c}, \end{array}$$

where $s_{i}^{c}$ is the complement set of $s_{i}$. Thus, the calculations essentially determine the probability of events being unobserved.

An actual experiment produces a finite set of putative observation events $O=\{\left[g_{j}\in s_{i}\right]:1\;\leq \;i\;\leq \;n;1\leq j\leq m\}$, from which a subset occurs ($\left[g_{j}\in s_{i}\right]$) and the complement does not ($\left[g_{j}\notin s_{i}\right]$).

#### Appendix B.1.2. Observation of Events in Merged Samples

The observation of events in merged samples is modelled by a two step procedure, and bases on two separate stochastically distributed entities:

- First, observation probabilities ($p_{j}$) are drawn from an observation prior Π.
- Second, the actual observation of events $\left[g_{j}\in s_{i}\right]$ are iid (independent identical distributed) drawn from a Bernoulli distribution $B(1,p_{j})$.

Thus, observation probabilities are distributed according to the observation prior Π. For a set of events G, a vector of observation probabilities $p=\left(p_{1},\ldots ,p_{m}\right)\in {\left[0,1\right]}^{m}$ is obtained the from the observation prior Π. The probability for observation of an event is given by

(A3) $$\begin{array}{c} P\left[g_{j}\in s_{i}\right]=E_{P}\left[1_{i}\left(g_{j}\right)\right]=:p_{j}, \end{array}$$

where P is the probability measure defined by $B(1,p_{j})$ on *O*, and $E_{P}$ is the expectation with respect to P. The number of observations in each sample is $|s_{i}|=\sum_{j=1}^{n}1_{i}\left(g_{j}\right)$ and the expectation for $|s_{i}|$ is given by

(A4) $$\begin{array}{c} E_{P}\left[\right|s_{i}\left|\right]=\sum_{j=1}^{m}E_{P}\left[1_{i}\left(g_{j}\right)\right]=\sum_{j=1}^{m}p_{j}. \end{array}$$

*Counting of observations in merged samples:* Observations $g_{j}\in s_{i}$ in a sample batch $B_{n}$ are counted using an indicator function

$$\begin{array}{c} 1_{S_{n}}\left(g_{j}\right):=\left\{\begin{array}{cc} 1 & \mathrm{when}\;g_{j}\;\in \;{⋃}_{i=1}^{n}s_{i}\\ 0 & \mathrm{when}\;g_{j}\;\notin \;{⋃}_{i=1}^{n}s_{i}. \end{array}\right. \end{array}$$

The second case ($g_{j}\;\notin \;\cup_{i=1}^{n}s_{i}$) is equivalent to $g_{j}\in \cap_{i=1}^{n}s_{i}^{c}$. Thus, $1_{S_{n}}\left(g_{j}\right)$ can be rewritten as

$$\begin{array}{c} 1_{S_{n}}\left(g_{j}\right)=1-\prod_{i=1}^{n}\left(1-1_{i}\left(g_{j}\right)\right). \end{array}$$

Using the independence of event observations in different samples, it follows that

(A5) $$\begin{array}{c} E_{P}\left[\prod_{i=1}^{n}\left(1-1_{i}\left(g_{j}\right)\right)\right]=\prod_{i=1}^{n}E_{P}\left[\left(1-1_{i}\left(g_{j}\right)\right)\right]={\left(1-p_{j}\right)}^{n} \end{array}$$

is the probability for not observing $g_{j}$ in $B_{n}$. Thus, the expectation for observation of an event $g_{j}$ in a batch $B_{n}$ is given by

(A6) $$\begin{array}{c} E_{P}\left[1_{i}\left(g_{j}\right)\right]=1-{\left(1-p_{j}\right)}^{n}. \end{array}$$

As the observation probabilities $p_{j}$ themselves are random variables, the expectation for event observation requires an additional integration with respect to the observation prior Π

(A7) $$\begin{array}{c} E_{\Pi }\left[g_{j}\in S_{n}\right]=\int 1-{\left(1-p_{j}\right)}^{n}\;d\Pi \left(p\right). \end{array}$$

The number of observed events in a merged sample

(A8) $$\begin{array}{c} |S_{n}|=\sum_{i=1}^{m}1_{S_{n}}\left(g_{j}\right) \end{array}$$

is a random variable, taking values in {0,…,m}. As the observation probabilities $P[g_{j}\in s_{i}]$ are equally distributed for all events, the expectation of the total number of events can simply be calculated from $E_{\Pi }\left[g_{j}\in S_{n}\right]$ using

(A9) $$\begin{array}{c} E_{\Pi }\left[\right|S_{n}\left|\right]=m\;E_{\Pi }\left[g_{1}\in S_{n}\right]=m\;\int 1-{\left(1-p\right)}^{n}\;d\Pi \left(p\right). \end{array}$$

#### Appendix B.1.3. Estimation of Observation Prior from Real Samples

The application of the model requires estimation of observation priors from real samples. The prior probabilities will be estimated from the multiplicity of observed events (*nProbes*: The number of samples in which a gap-site is identified) and thus bases on count data.

*Discretised integration:* Assume, that Π is a discrete measure. Let $p_{\Pi }=\left(p_{1},\ldots ,p_{k}\right)$ be a set of distinct observation probabilities, which shall occur with prior probabilities $\pi =(\pi_{1},\ldots ,\pi_{k})$. The integral in Equation (A9) then transforms into a sum

(A10) $$\begin{array}{c} E_{\Pi }\left[\right|S_{n}\left|\right]=m\left(1-\sum_{j=1}^{k}{\left(1-p_{j}\right)}^{n}\pi_{j}\right). \end{array}$$

*Empirical estimates for observation probabilities:* Recall $|S_{n}|$, the number of observed events in a batch $B_{n}$ of samples. The multiplicities are numbers in {1,…,n} and the estimated observation probabilities thus are

(A11) $$\begin{array}{c} \hat{p}=\left\{\frac{1}{n},\ldots ,\frac{n}{n}\right\}. \end{array}$$

For k∈{1,…,n}, let $z_{k}$ the number of events observed with multiplicity *k*. The vector $Z=\{z_{1},\ldots ,z_{n}\}$ then is a vector of count values, from which estimations for the prior probabilities $\hat{\pi }=\left(\hat{\pi_{0}},\ldots ,\hat{\pi_{n}}\right)$ are calculated using the definition

(A12) $$\begin{array}{c} \hat{\pi_{j}}=\frac{z_{j}}{|S_{n}|}. \end{array}$$

*Estimation of number of observed events:* Let ν∈{1,…,n}. We want to estimate the number of observed events in a subset of samples ($S_{\nu }\subset B_{n}$). Using the discretised prior, the model derived estimation for the total number of observed events in $S_{\nu }$ is given by

(A13) $$\begin{array}{c} E_{\Pi }\left[\right|S_{\nu }\left|\right]=\;m\;\left(1-\sum_{j=1}^{n-1}{\left(1-\hat{p_{j}}\right)}^{\nu }\hat{\pi_{j}}\right). \end{array}$$

By replacing the total number of observable events (*m*) by the total number of observed events ($|S_{n}|$), the final estimate for the number of observed events in a batch of size ν,

(A14) $$\begin{array}{c} \hat{|S_{\nu }|}=|S_{n}|\left(1-\sum_{j=1}^{n-1}{\left(1-\frac{j}{n}\right)}^{\nu }\frac{z_{j}}{|S_{n}|}\right)=\left|S_{n}\right|-\sum_{j=1}^{n-1}{\left(1-\frac{j}{n}\right)}^{\nu }z_{j} \end{array}$$

is obtained.

### Appendix B.2. Evaluation of Model Predictions for Special Cases

The following section evaluates model-based predictions for selected special cases. Although, in transcriptome data, these specialisations are not isolated observed but part of a heterogeneous mixture of events, they provide insight into relationships between observation probabilities and sample composition.

#### Appendix B.2.1. Equal Observation Probabilities

We assume, that observation probabilities are equal for all events ($p_{j}=p<1\;\forall j$). Thus only one observation prior $\pi_{1}=1$ exists and Equation (A7) reduces to

(A15) $$\begin{array}{c} E_{\Pi }\left[g_{j}\in S_{n}\right]=1-{\left(1-p\right)}^{n}. \end{array}$$

The expected total number of events thus is

(A16) $$\begin{array}{c} E_{\Pi }\left[\right|S_{n}\left|\right]=m\;(1-{\left(1-p\right)}^{n}). \end{array}$$

#### Appendix B.2.2. Events Observed in All Samples

When all events $g_{j}$ are observed in all samples, meaning that p=1 in Equation (A16), the expected number of events is to $E_{\Pi }\left[\right|S_{n}\left|\right]=m$. The observed number of events is |G| (the number of observable events) independent from *n*.

#### Appendix B.2.3. Observation of Rare Events

When a small observation probability $p_{j}\ll 1$ is assumed for all events $g_{j}$, so that ${\left(1-p_{j}\right)}^{n}$ can be approximated by the first Taylor expansion (${\left(1-p_{j}\right)}^{n}\approx 1-np_{j}$), the expected total number of events becomes

(A17) $$\begin{array}{c} E_{\Pi }\left[\right|S_{n}\left|\right]=m\;n\sum_{j=1}^{k}p_{j}\pi_{j}. \end{array}$$

Thus, a linear increase of the number of observed events ($|S_{n}|$) with *n* (the number of samples), is observed. As this observation rate provides a theoretical upper limit, the real number of events increases sub-linear with *n*.

#### Appendix B.2.4. Rare Events Observed with Equal Probability:

Additionally, when all events are observed with equal observation probability p≪1, Equation (A17) transforms to $E_{\Pi }\left[\right|S_{n}\left|\right]=m\times p\times n$. The fact, that the observation rate only allows estimation of m×p, but not *m* or *p* directly, imposes uncertainties on the absolute size of *m*.

## Appendix C. Gap-Sites Identified in Only One Sample

Gap-sites present in only one sample are called “unique” gap-sites. Their number may increase or decrease when new samples are added to a batch.

The distribution of unique gap-sites in fibroblast samples from TopHat and STAR is shown in Figure A2. The number of unique gap-sites varies substantially between samples in alignments from one aligner (TopHat or STAR). In alignments from TopHat, in total 384,576 unique gap-sites are identified (mean 7122, SD 2905 per sample). In alignments from STAR, in total 4,655,597 unique gap-sites are identified (mean 86,215, SD 34,464 per sample).

## Appendix D. Derivation of of the Formula for Sample Size Estimation

A derivation for the provided formula for sample size estimation (Equation (3)) is given here. Denote the lowest observation probability for an event of interest as $p_{g}$. Equation (A6) then provides the probability of being observed in a batch of size *n*: $p_{b}=1-{\left(1-p_{g}\right)}^{n}$. The algebraic reformation

(A18) $$\begin{array}{ccc} \left(1-p_{b}\right) & ={\left(1-p_{g}\right)}^{n}=e^{n\times ln(1-p_{g})} & \Leftrightarrow \end{array}$$

(A19) $$\begin{array}{ccc} ln(1-p_{b}) & =n\times ln(1-p_{g}) & \Leftrightarrow \end{array}$$

(A20) $$\begin{array}{ccc} n & =\frac{ln(1-p_{b})}{ln(1-p_{g})} & \end{array}$$

then allows direct calculation of *n*, when the desired power (for example 80%) is introduced as value for $p_{b}$.
